# AMMI and GGE biplot analysis of genotype by environment interaction for yield and yield contributing traits in confectionery groundnut

**DOI:** 10.1038/s41598-024-52938-z

**Published:** 2024-02-05

**Authors:** Praveen Kona, B. C. Ajay, K. Gangadhara, Narendra Kumar, Raja Ram Choudhary, M. K. Mahatma, Sushmita Singh, Kiran K. Reddy, S. K. Bera, Chandramohan Sangh, Kirti Rani, Zarana Chavada, K. D. Solanki

**Affiliations:** 1 ICAR-Directorate of Groundnut Research, Junagadh, Gujarat 362001 India; 2 RRS, ICAR-Directorate of Groundnut Research, Ananthapur, Andhra Pradesh India; 3 RRS, ICAR-Central Tobacco Research Institute, Kandukur, Andhra Pradesh India; 4 RRS, ICAR-Directorate of Groundnut Research, Bikaner, Rajasthan India; 5 ICAR-National Research Centre on Seed Spices, Ajmer, Rajasthan India; 6 ICAR-National Bureau of Plant Genetic Resources Regional Station, Jodhpur, Rajasthan India

**Keywords:** Plant sciences, Plant breeding

## Abstract

The global market has a high demand for premium edible grade groundnut, particularly for table use. India, in particular, exhibits significant potential for exporting confectionary grade large seeded groundnut. The environment plays a significant impact in influencing the expression of seed traits, which subsequently affects the confectionary quality of groundnut genotypes. The states of Gujarat and Rajasthan in India are prominent producers of high-quality groundnuts specifically used for confectionary purposes. The current study was conducted with 43 confectionery groundnut genotypes at Junagadh, Gujarat, and Bikaner, Rajasthan, with the goals of understanding genotype-by-environment interaction (GEI) effects and identifying stable, high yielding confectionery quality groundnut genotypes using AMMI and GGE biplot models. Pod yield per plant (PYP), number of pods per plant (NPP), hundred kernel weight (HKW), and shelling percent (SP) were estimated. The interplay between the environment and genotype has had a notable impact on the manifestation of confectionary grade characteristics in peanuts. The results from the Interaction Principal Component Analysis (IPCA) indicate that HKW contributed 76.68% and 18.95% towards the Global Environmental Index (GEI) through IPCA1 and IPCA2, respectively. Similarly, NPP contributed 87.52% and 8.65%, PYP contributed 95.87% and 2.1%, and SP contributed 77.4% and 16.22% towards GEI through IPCA1 and IPCA2, respectively. Based on the ranking of genotypes, the ideal genotypes were PBS 29079B for HKW, PBS 29230 for NPP. The genotypes PBS 29233 and PBS 29230 exhibited superior performance and stability in terms of pod yield, hundred kernel weight, number of pods per plant, and shelling percentage across various sites. These breeding lines have the potential to be developed for the purpose of producing confectionary grade groundnut with larger seeds, in order to fulfil the growing demand for export.

## Introduction

Groundnut, *Arachis hypogaea L*., is a major oilseed crop worldwide. It has a unique nutritional profile and has long been used in cooking^1^. Groundnut also provides dietary fibre, B group vitamins, vitamin E, and minerals like iron, zinc, potassium, and magnesium^2^. Trace minerals including selenium, manganese, and copper enhance cellular functioning, as can antioxidant polyphenolic substances like flavonoids and resveratrol^3,4^.

The global market for premium confectionery peanuts is large. India has abundant confectionery and large-seeded groundnut export potential. Groundnut seed quality depends on physical, sensory, chemical, and nutritional factors. New groundnut cultivars must have specified physical and chemical characteristics, processing, and end-use properties to be accepted by merchants, manufacturers, and consumers^5,6^. High standards for sound mature kernels (SMK), 100-kernel weight (HKW), elongated shape, tapering ends, and pink to light brown testa colour are desirable^7^. Groundnut's consumable value depends on seed size and chemical composition. Few genotypes have been selectively bred for Hand Picked Selection (HPS) acceptance.

Genotype environment interactions (GEI) affect confectionery (i.e. HKW) and other yield-related traits like pod yield, shelling percent (SP), and number of pods per plant (NPP) genotypes, which perform differently in different environments. Under such conditions, stability analysis using multi-environment trials (METs) works well to evaluate genotype performance over environments. The additive main effects and multiplicative interaction (AMMI) model and genotype plus genotype environment interaction (GGE) biplot are two statistical methods for studying gene-environment interaction (GEI) effects^8,9^. Both methods use two-way environment tables and principal component analysis (PCA) to visualise complex genotypes^10^. How the means are treated before singular value decomposition distinguishes the two approaches. Unlike GGE biplot, AMMI applies SVD to GE interaction data without genotype and environment means^11^. Due to their excellent correlation, both approaches can be used interchangeably^12^.

The GGE biplot uses mean vs. stability plots, test environment evaluation using discriminating power vs. representativeness plots, and multi-environment analysis such "which-won-where" pattern identification^13,14^. Since its introduction, the GGE biplot technique has been used in MET analysis many times. Previous research used GGE biplot analysis to evaluate 24 BGN landrace yield stability^15^. The yield stability of Andean dry bean accessions grown under varied abiotic stress conditions in Tanzania was studied using GGE biplot analysis^16^. This study's main objectives were to (i) characterise groundnut genotypes at two locations in terms of seed size and yield-related traits, and (ii) assess genotype-by-environment interaction and identify stable genotypes with large seed size and yield-related traits in two prominent groundnut growing regions of India.

## Materials and methods

### Study locations

**Graph 1:**
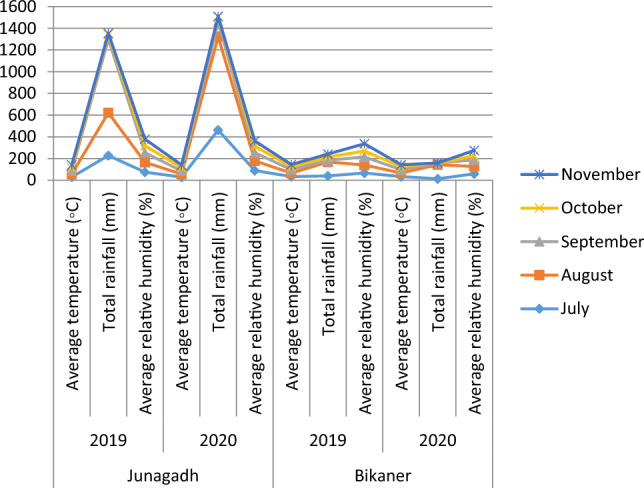
Graphical representation of monthly meteorological data of the experimental sites during groundnut growing seasons.

The study was conducted in two discrete agroecological zones, specifically Junagadh, Gujarat and Bikaner, Rajasthan, throughout the *Kharif* seasons of 2019 and 2020. Table 1 and Graph 1 display the average meteorological data pertaining to the locations under investigation.

**Table 1:** Monthly meteorological data of the experimental sites during groundnut growing seasons.

Location	Latitude	Longitude	Year	Parameter	July	August	September	October	November
Junagadh	28.075758	73.345096	2019	Average temperature (°C)	30.2	27.8	28.1	28.8	26.6
				Total rainfall (mm)	228.2	393.8	678.7	42.2	11.2
				Average relative humidity (%)	75	90	88	65	61
			2020	Average temperature (°C )	29.1	27.8	29.2	29.6	25.1
				Total rainfall (mm)	462.6	862.0	136.1	48.9	0.0
				Average relative humidity (%)	89	90	76	59	48
Bikaner	21.506323	70.449339	2019	Average temperature (°C )	34.3	31.5	32	26.6	20
				Total rainfall (mm)	40.6	128.2	16.2	28.8	27.2
				Average relative humidity (%)	66.3	74.1	74.2	55.6	66.4
			2020	Average temperature (°C )	34.3	32.3	31.2	26.1	19.0
				Total rainfall (mm)	13.4	128.9	15.6	0.0	1.2
				Average relative humidity (%)	59.2	68.1	60.9	37.9	48.9

### Soil sampling and analysis

A comprehensive soil sample was obtained from the entire plot by collecting samples from a depth range of 0–15 cm utilising a soil auger. The composite sample was thereafter employed for the initiation of experiment following the completion of the harvest. The soil sample underwent shade drying and subsequent sieving using a 2 mm sieve in order to facilitate chemical testing, which encompassed the determination of sand, clay, silt, pH, organic carbon (OC), total nitrogen (N), potassium (K), accessible phosphorus (P), and particle size distribution. The aforementioned analyses were conducted at the commencement of the trial.

### Plant materials and data collection

A comprehensive evaluation was conducted on 43 groundnut genotypes during the *Kharif* seasons of 2019 and 2020. The assessment was carried out using a randomised complete block design, with three replicates. The dataset comprised of 40 breeding lines that were at an advanced stage of development, along with three check varieties: Girnar 2, Mallika, and Raj Mungfali 3 (Table 2). Each plot was measured to have a length of 3 m, with a plant spacing of 10 cm and a row spacing of 60 cm between each plot. The seeding process occurred during the first two weeks of July, while the harvesting activities were conducted during the first two weeks of November. In order to develop a successful crop, strict adherence to the defined set of practises was observed. The researchers recorded data related to pod production, shelling percentage (SP), number of pods per plant (NPP), and hundred kernel weight (HKW). The process of measuring Hundred Kernel Weight (HKW) entailed the random selection of mature hundred seeds from each genotype within each replication. The seeds that were chosen were then quantified in grams. The shelling percentage (SP) was ascertained by computing the proportion of the weight of seeds obtained from the sample of pods to the overall weight of the pods, denoted as a percentage. The mature pods from each individual plant were separated and afterwards subjected to a drying process for a duration of seven days, until they attained a moisture content that confirmed to established standards. Following this, the specimens underwent a cleaning process and were subsequently weighed via a balance instrument. The weight of the pods was afterwards divided by the plant population in order to ascertain the pod yield per plant. In a similar manner, the total number of pods was quantified and afterwards divided by the plant population in order to get the average number of pods per plant.

**Table 2:** Average performance of genotypes for yield related traits at Junagadh and Bikaner locations.

S. No	Genotype	HKW		NPP		PWP		SP	
		Jun	Bik	Jun	Bik	Jun	Bik	Jun	Bik
G1	GIRNAR 2 (C1)	43.47	61.69	15.59	26.77	9.81	29.36	60.19	71.6
G2	Mallika (C2)	48.59	83.8	13.57	14.23	10.2	27.23	62.24	62.44
G3	PBS 19013	25.14	66.62	9.5	14.38	6.72	18.56	54.99	60.06
G4	PBS 19015	43.32	70.25	10.64	12.8	7.91	13.92	55.64	63.24
G5	PBS 19018	37.7	74.6	12.39	19.57	7.39	29.61	48.41	63.48
G6	PBS 19029	47.01	87.13	10.64	10.9	9.6	18.76	52.65	60.39
G7	PBS 29069	34.94	71.4	12.25	10.77	8.93	14.74	63.15	51.76
G8	PBS 29078	48.74	101.78	10.94	14.03	10.05	23.17	56.72	65.35
G9	PBS 29079A	54.81	72.73	10.14	12.15	6.91	18.29	55.92	59.16
G10	PBS 29079B	72.53	121.95	9.73	21.13	7.52	40.06	79.84	62.2
G11	PBS 29082	44.49	64.6	11.43	11.18	7.2	16.12	46.73	47.45
G12	PBS 29137	45.32	88.98	14.46	12.93	14.73	20.2	54.26	60.62
G13	PBS 29138	46.92	89.57	11.28	13.7	7.09	23.35	53.76	63.97
G14	PBS 29143	42.2	91.31	11.44	10.9	11.2	17.83	45.3	60.97
G15	PBS 29160	44.75	88.72	12.62	18.87	9.28	33.55	59.44	64.55
G16	PBS 29165	45.55	83.24	7.06	11.8	6.72	16.52	60.7	64.93
G17	PBS 29167	44.54	94.84	11.1	13.75	9.88	23.48	56.69	68.16
G18	PBS 29187	43.46	84.92	9.26	12.4	8.1	19.13	56.07	65.79
G19	PBS 29189	40.49	76.41	12.69	11	10.96	12.52	63.27	66.33
G20	PBS 29191	52.57	106.52	11.52	14.47	8.94	27.87	59.25	67.76
G21	PBS 29193	54.78	71.43	10.03	10.77	8.35	13.08	54.38	54.8
G22	PBS 29194	58.08	70.25	10.74	10.93	8.73	14	54.38	54.27
G23	PBS 29197	40.71	104.1	10.72	12.67	9.26	26.05	57	67.33
G24	PBS 29199	48.78	86.21	9.63	13.53	7.65	22.47	58.43	65.3
G25	PBS 29204	50.53	86.44	10.09	14.4	5.16	19.1	54.96	59.09
G26	PBS 29207	50.28	104.05	8.66	11.45	6.59	23	56.09	67.84
G27	PBS 29208	55.55	100.22	12.07	17.53	9.01	36.65	56.78	67.7
G28	PBS 29210	58.09	98.57	11.62	17.43	9.22	27.2	57.72	65.97
G29	PBS 29211	47.01	100.41	10.92	14.6	8.96	31.01	57.07	73.27
G30	PBS 29212	47.14	83.59	7.02	10.5	6.56	13.98	54.27	58.61
G31	PBS 29214	55	99.52	10.65	13.33	7.43	28.63	55.76	64.97
G32	PBS 29218	57.99	102.19	9.07	18.77	6.35	33.76	51.61	67.97
G33	PBS 29219	60.79	90.69	12.35	16.6	9.05	23.59	48.78	56.9
G34	PBS 29223	54.62	83.48	13.24	15.63	9.55	26.12	51.32	60.96
G35	PBS 29228	56.36	67.17	12.69	12.37	8.65	16.46	59	65.51
G36	PBS 29232	47.34	62.23	16.33	17.7	8.18	21.52	61.38	72.85
G37	PBS 29233	46.06	67.76	15.06	19.07	10.69	27.64	61.91	71.36
G38	PBS 29243	44.69	70.53	11.44	15.73	7.89	22.03	59.05	65.53
G39	PBS 29192	54.84	91.1	10.73	13.37	8.59	25.78	58.03	70.55
G40	PBS 29195	54.03	74.97	10.6	10.73	9.44	14.04	55.31	53.16
G41	PBS 29225	55.9	100.57	11.22	14.47	9.35	27.19	57.73	68.33
G42	PBS 29230	50.15	66.56	17.54	21.5	10.58	24	61.02	70.11
G43	Raj Mungpali 3 (C3)	43.22	97.3	10.79	14.5	9.15	27.42	58.19	67.23
	CV (%)	1.90	1.1	4.45	3.21	5.45	3.43	1.45	0.96

### Material statement

Test materials were sourced from our own gene bank at ICAR-Directorate of Groundnut Research, Junagadh. All the plant material was obtained and developed at ICAR-DGR, Junagadh and no specific permissions are required as they are our own material.

### Statistical analysis

The data analysis was performed using version 4.2.1 of the R statistical software^17^. The data on yield and yield-related variables were analysed using a combined analysis of variance (ANOVA) to evaluate the existence of genotype by environment interaction (GEI). The data underwent log transformation for normalisation prior to analysis. In order to facilitate AMMI and GGE biplot modelling, each year and location was considered as a distinct and autonomous environment and were analysed using packages "agricolae"^18^ and GGEBiplotGUI^19^ respectively.

#### Handling plant materials and methods

The collection and handling of plant and methods were in accordance with all the relevant guidelines.

## Results and discussion

### Soil analysis

The physicochemical parameters of the soil for the two specified areas are presented in Table 3. According to the data presented in Table 3, it can be observed that Junagadh demonstrates somewhat elevated concentrations of nitrogen, organic carbon, phosphorus, and potassium in comparison to Bikaner. The soil characteristics observed in Bikaner indicate significantly greater levels of bulk density and pH when compared to Junagadh. Significant differences in soil parameters were observed at both study sites throughout the course of the two-year period, which had a notable influence on crop productivity and related attributes. The productivity of groundnut is found to be higher in sandy soils compared to clayey soils, mostly due to the porous structure of sandy soils that enables pod expansion. The occurrence of slender and loosely connected fissures in sandy soils during the process of desiccation is a desirable attribute, especially in areas characterised by semi-arid climates, where rainfall patterns are unpredictable and the soil undergoes prolonged periods of dryness. Although clay soil has a notable ability to hold water, it experiences an increase in volume when it becomes wet and a decrease in volume when it dries over long periods of time.

**Table 3:** Characterization of soil properties of experimental locations.

Location	PH	Texture	Bulk density	OC (%)	N (Kg/ha)	P (Kg/ha)	K (Kg/ha)
Bikaner	8.30	Loamy sand	1.55	0.15	92.26	14.62	207
Junagadh	7.85	Clayey	1.34	0.62	235	30	315

### Combined analysis of variance

A thorough examination was conducted (as illustrated in Table 4) to deconstruct the primary impacts and evaluate the interconnections among and within the factors of variation. The study's results revealed significant variations in yield (PYP) and related traits (HKW, NPP, SP) as a result of the combined effects of genotypes, seasons, and environments, as well as the interactions between genotype and environment (GEI). These interactions encompassed location × Season, location × Genotype, Season × Genotype, and location × Season × Genotype. The findings of this study suggest that there were variations in the performance of accessions across different locations and years, primarily attributed to differences in environmental conditions. These disparities subsequently altered the expression of genotypes in diverse settings and seasons. Furthermore, it should be noted that the impact of the environment on genotypes is not consistent, as different genotypes display distinct responses in terms of yield and associated traits under different environmental circumstances. This, phenomena has been extensively observed by several authors^20–28^.

**Table 4:** Combined pooled Analysis of variance for yield and yield related traits data obtained from trials conducted in Bikaner and Junagadh in 2019 and 2020 (environments constitute year–location combinations).

EFFECT	DF	HKW			NPP			PYP			SP		
		SS	MS	%SS	SS	MS	%SS	SS	MS	%SS	SS	MS	%SS
Rep	2	2.02	1.01	0.001	3.51	1.75**	0.06	0.36	0.18	0.0009	1.01	0.50	0.004
Environment (L)	1	170202.79	170202.79**	68.63	1250.25	1250.25**	20.95	26381.36	26381.36**	66.67	6047.73	6047.73**	24.72
Season (S)	1	510.38	510.38**	0.20	49.86	49.86**	0.83	377.36	377.36**	0.95	502.00	502.00**	2.05
Genotype (G)	42	46802.85	1114.35**	18.87	3191.22	75.98**	53.46	6099.85	145.23**	15.41	10593.87	252.23**	43.3
L × S	1	458.95	458.95**	0.18	44.89	44.89**	0.75	416.29	416.29**	1.05	592.55	592.55**	2.42
G × L	42	22281.81	530.52**	8.98	1132.27	26.96**	18.97	5960.76	141.92**	15.06	5354.32	127.48**	21.89
G × S	42	3915.15	93.22**	1.57	108.73	2.59**	1.82	121.75	2.90**	0.31	520.58	12.39**	2.13
G × L × S	42	3523.17	83.89**	1.42	99.43	2.37**	1.67	64.20	1.53**	0.16	653.38	15.56**	2.67
Residual	342	304.73	0.89	0.12	88.48	0.26	1.48	144.22	0.42	0.36	196.87	0.58	0.80
Total	515	248001.83			5968.65			39566.15			24462.29		

Coefficient of variation = 0.39. *DF* = Degree of freedom. *SS* = sum of squares. *MS*. = mean square. *Significant at *p* ≤ 0.05. **Significant at *p* < 0.001.

### Additive main effects and multiplicative interaction1 biplot

**Figure 1:**
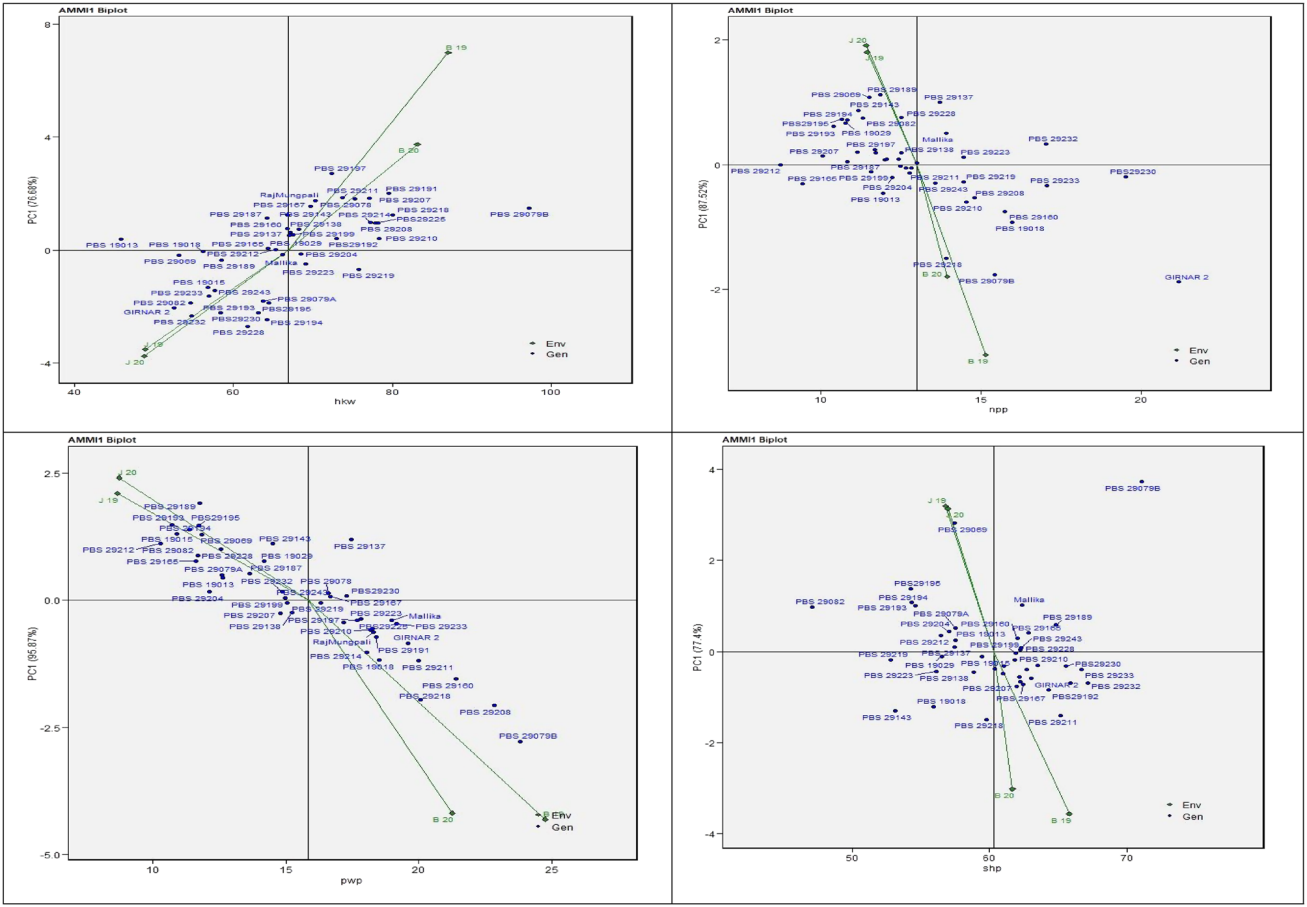
Additive main effects and multiplicative interaction 1 (AMMI 1) biplots based on PC1 illustrating G × E interactions of the 43 groundnut accessions in two seasons and two locations: (**A**) Hundred kernel weight (HKW) (**B**) No. of pods per plant (NPP), (**C**) pod yield per plant (PWP), (**D**) Shelling percent (SP).

The use of the additive main effects and multiplicative interaction (AMMI) model is a prominent statistical methodology for investigating genotype by environment interaction (GEI) and genotype stability. The graphical representation in Fig. 1 depicts the abscissa and ordinate of the AMMI 1 biplot. These axes are associated with the first Interaction principal component (IPCA1) term and the primary impacts of the traits HKW (Fig. 1A), NPP (Fig. 1B), PYP (Fig. 1C), and SHP (Fig. 1D). The IPCA1 scores were shown in correlation with these variables across all contexts, providing useful insights into the genotype-environment interaction of the studied genotypes. The analysis performed by the GEI revealed both similarities and variations across the 43 genotypes, as evidenced by the IPCA1 values of 76.68% for HKW, 87.52% for NPP, 95.87% for PYP, and 77.4% for SP. For HKW (as seen in Fig. 1A), it was observed that genotypes PBS 29212 (G30), PBS 19018 (G5), and PBS 29165 (G16) exhibited significant stability. The proximity of the data points to the origin and their near-zero values on the IPCA1 were indicative of this. The observed genotypes exhibit significant adaptability and possess favourable characteristics for achieving hundred kernel weight (HKW) under diverse environmental conditions. On the other hand, it is noteworthy that the genotypes PBS 29228 (G35), PBS 29197 (G23), PBS 29079B (G10), and PBS 29191 (G20) exhibited unstable characteristics, as evidenced by their considerable deviation from the reference point. These genotypes also demonstrated a constrained capacity for adaptation and are better suited for environments characterised by limited conditions. The genotype PBS 29228 (G35) exhibited restricted adaptability to environmental conditions in Junagadh, whereas the genotype PBS 29297 (G23) exhibited adaptability to Bikaner. Figure 1B illustrates that the genotypes PBS 29138 (G13) and PBS 29211 (G29) exhibited minimal scores on the IPCA1. The genotypes examined in this study were found to be located in close proximity to the origin, suggesting that they possess a high degree of adaptation in relation to number of pods per plant (NPP). On the other hand, it is worth noting that Girnar 2 (G1), PBS 29079B (G10), PBS 29230 (G42), PBS 29232 (G36), PBS 29189 (G19), and PBS 29212 (G30) exhibited a considerable spatial separation from the place of origin and displayed restricted levels of adaptation. In relation to the pod yield per plant (PYP) as illustrated in Fig. 1C, it was seen that PBS 29219 (G33), PBS 29199 (G24), PBS 29167 (G17), and PBS 29078 (G8) demonstrated significant adaptation, as evidenced by their closeness to the origin and their virtually zero value on the IPCA1. On the other hand, PBS 29079B (G10), PBS 29189 (G19), PBS 29208 (G27), and PBS 29218 (G32) demonstrated a significant degree of spatial divergence from the reference point and exhibited a restricted scope of adaptability. Genotypes PBS 29079B (G10), PBS 29208 (G7), and PBS 29160 (G15), which were positioned on the right side of the central axis, exhibited increased production. In relation to Shelling percent (SP) as illustrated by Fig. 1D depicts the proximity of PBS 19015 (G4), PBS 29199 (G24), and PBS 29210 (G28) to the origin, indicating that they exhibit low scores on the first interaction principal component (IPCA1). On the contrary, PBS 29079B (G10), PBS 29069 (G7), PBS 29082 (G11), and PBS 29211 (G29) exhibit a substantial spatial separation from the point of origin.

In the context of the AMMI1 investigation, it has been observed that genotypes located near the centre region of the IPCA1 axis exhibit greater stability with minimal interaction effects. As a result, these genotypes demonstrate a wide range of adaptation to various environmental situations. In contrast, the detection of a positive interaction occurs when there is congruent polarity between the genotype and environment on the IPCA axis. Conversely, a deleterious interaction arises when there is a discrepancy in polarity between the genotype and the environment. The outcomes derived from our research are consistent with the findings documented^29–33^. The findings of present investigation demonstrate that the AMMI model exhibits a satisfactory level of compatibility with the collected data, thereby providing empirical justification for the application of AMMI 2. The scholarly works of^34–37^ have been essential in facilitating the creation of the biplot and the calculation of genotype and environment effects.

### Additive main effects and multiplicative interaction 2 biplot

**Figure 2:**
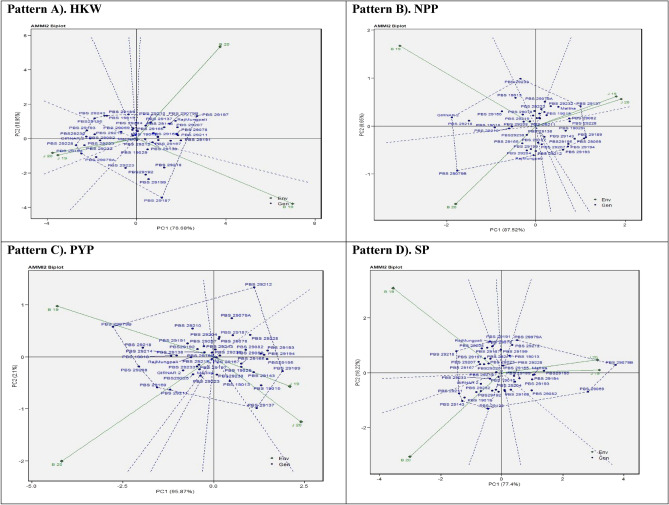
Additive main effects and multiplicative interaction 2 (AMMI 2) biplots based on PC1 illustrating G × E interactions of the 43 groundnut accessions in two seasons and two locations: (**A**) Hundred kernel weight (HKW) (**B**) No. of pods per plant (NPP), (**C**) pod yield per plant (PWP), (**D**) Shelling percent (SP).

The findings derived from the AMMI 2 analysis have yielded support for the significance of including IPCA2 scores in conjunction with IPCA1 scores to enhance our understanding of genotype-environment interactions (GEI) across diverse settings. Moreover, the utilisation of this methodology has facilitated the identification of genotypic adaptations, as depicted in Fig. 2. The values of IPCA2 index for four variables, namely HKW, NPP, PYP, and SP, which were found to be 18.95%, 8.65%, 2.1%, and 16.22% respectively. The findings of the study indicate that the initial two IPCAs were responsible for the entirety of the Genotypic-by-Environmental Interaction (GEI) variance across the four variables that were investigated. The outcomes of our investigation are consistent with the findings^34^, who noted that the initial two primary components adequately serve as a basis for projecting the AMMI model. It is important to acknowledge that^38^, have proposed the incorporation of the initial four primary components within the framework of multi-environment trials. The AMMI2 biplot use the distances from the biplot origin to provide insights into the degree of interaction exhibited by genotypes across environments, or conversely. Environments with low IPCA1 and IPCA2 scores, located near the origin, have a notable influence on genotype stability was reported^39^. Moreover, these habitats exhibit a constrained impact on genotype-by-environment interaction, implying a broad adaptability to diverse developmental circumstances.

Within the framework of HKW, it was noted that genotypes PBS 29212 (G30), PBS 19018 (G5), Mallika (G2), and PBS 29219 (G33) exhibited a significant proximity to the origin. In contrast, it was observed that genotypes PBS 29197 (G23), PBS 29187 (G18), and PBS 29199 (G24) exhibited a spatial distribution that deviated from the central region. In the context of NPP, it was found that certain genotypes, including PBS 29208 (G27), PBS 19018 (G5), PBS 29210 (G28), and PBS 29211 (G29), demonstrated a close proximity to the origin. However, genotypes PBS 29079B (G10) and PBS 29233 (G37) revealed a significant distance from the centre. The interaction between genotypic and the environment was observed to be highly impacted by a range of environmental variables. Within the framework of NPP, it was observed that PBS 29079B (G10) demonstrated significant adaptability towards B20. Conversely, PBS 29137 (G12) exhibited compatibility with J19 and J20, while PBS 29233 (G37) displayed a favourable response to B20. In the context of PYP, it was observed that genotypes PBS 29167, PBS 29232, PBS 29199 (G24), PBS 19018 (G5), and Raj Mungfali 3 (G43) demonstrated proximity to the origin. In contrast, genotypes PBS 29137 (G12), PBS 29212 (G30), and PBS 29079B (G10) exhibited a significant deviation from the central position. The genotype PBS 29079B (G10) displayed adaptability to B19, whereas PBS 29137 (G12) indicated remarkable adaptability specifically with J20. Furthermore, PBS 19015 (G4) and PBS 29189 (G19) exhibited a notable degree of flexibility in relation to J19. In relation to the percentage of shelling, it was noted that genotypes PBS 29195 (G40), Mallika (G2), PBS 29219 (G33), and PBS 29165 (G16) demonstrated a close proximity to the origin. In contrast, genotypes PBS 29138 (G13) and PBS 29079A (G9) exhibited a notable degree of separation from the central point. Furthermore, it was observed that PBS 29079B (G10) exhibited clear indications of adaptation when combined with J19 and J20.

The environments B19 and B20, which exhibit longer vectors, demonstrated a heightened level of interactivity and a superior capacity to detect changes across genotypes in relation to HKW, NPP, PYP, and SHP, in contrast to the J19 and J20 environments, which possessed shorter vectors. The results of the present investigation are consistent with the observations made by^40^, who indicated that genotypes located in closer proximity to the centre of the AMMI 2 model biplot tend to exhibit more stability^41^. has documented similar results in their respective studies.

### GGE biplot

The GGE biplot model serve as a valuable tool for comprehending the impacts of genotype by environment interaction (GEI) and identifying genotypes that exhibit adaptation to certain environments. It acknowledges the inherent limitations of genotypes, as they may not consistently excel across all situations. The GGE-biplot method is highly appropriate for analysing datasets that encompass multiple environments. This analysis can be facilitated by utilising various packages, such as the "which-won-where" pattern package, which aids in determining the discriminating ability and representativeness of environmental evaluations. Additionally, the genotypic evaluation can be conducted by assessing the mean performance and stability across different environments^27^.

#### ‘Discriminativeness vs. representativeness’ pattern of GGE biplot

**Figure 3:**
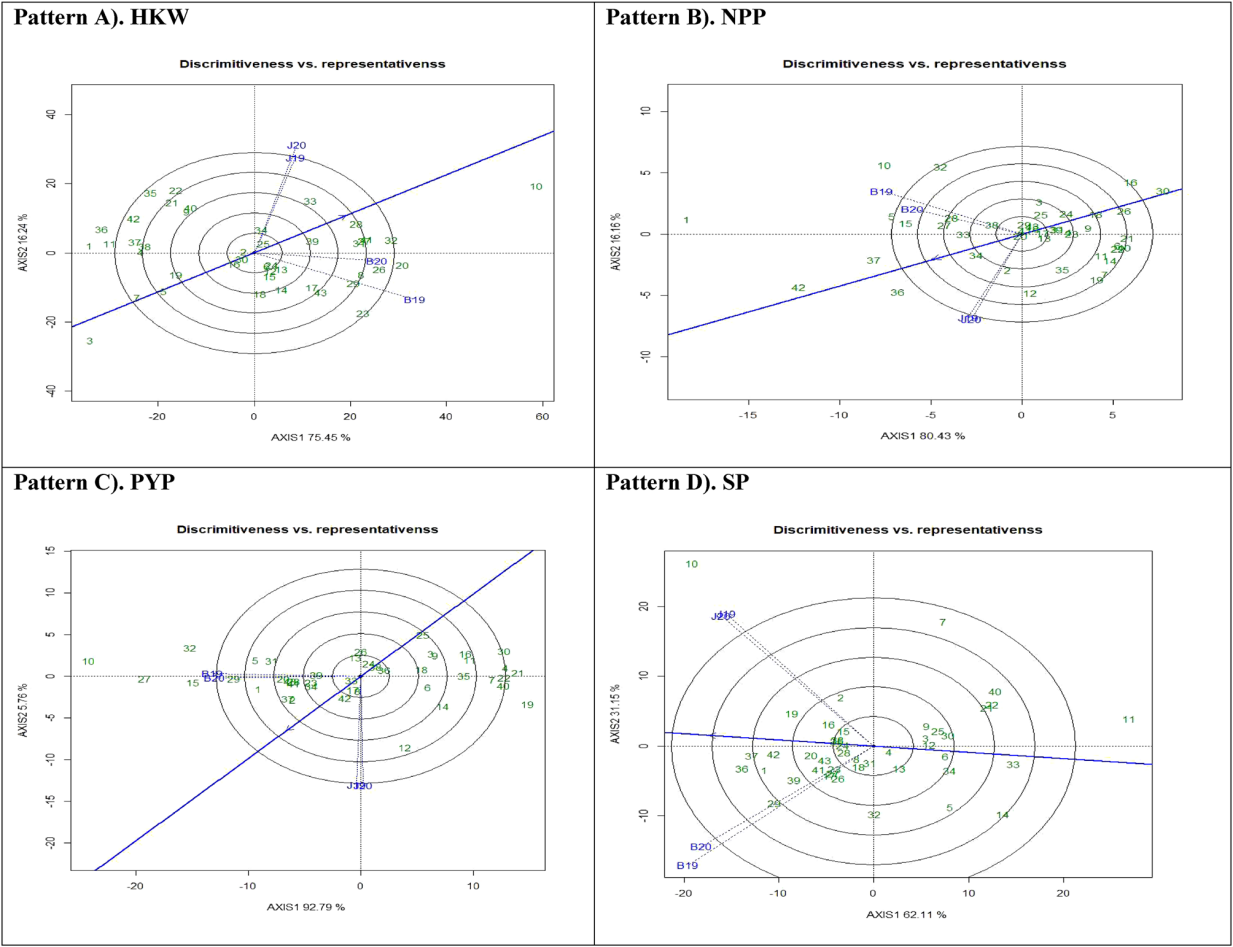
Patterns (A–D). Ranking genotypes based on PC1 and PC2 showing G × E interactions of the 43 groundnut accessions under two locations and two seasons: (**A**) Hundred kernel weight (HKW), (**B**) No. of pods per plant (NPP), (**C**) Pod yield per plant (PYP), (**D**) Shelling percent (SP).

Selection of an optimal test environment is of utmost importance in the implementation of a successful breeding strategy that results in the identification and cultivation of superior genotypes. Figure 3 depicts the ‘Discriminativeness vs. representativeness' view of GGE biplots for four traits being investigated, denoted as pattern A, B, C, and D. The vector length associated with each environment provided insight into the discriminatory capacity of the environment, while the angle produced by each vector with the abscissa indicated the level of representativeness. Environments characterised by longer vectors demonstrate a greater propensity for classifying genotypes in comparison to environments characterised by shorter vectors. According to previous studies^42^ it can be inferred that a test environment will be more representative when the angle is less.

The vector with the smallest magnitude among Patterns A, B, C, and D was B20, whereas the vector with the largest magnitude was seen for B19. The measurements of angles formed by environment in relation to the abscissa line were also documented. In the context of HKW, NPP, PYP, and SP, it was observed that the angle formed by B20 was the shortest, while the angles formed by B19 were found to be the longest. Nevertheless, an environment characterised by a longer vector that creates a smaller angle with the AEC abscissa line is considered optimal for the identification and selection of superior genotypes. Among the various patterns, namely A, B, C, and D, it was observed that J20 exhibited a tiny angle in conjunction with a lengthy vector, where the AEC abscissa indicated that the test environment was both representative and capable of distinguishing itself from other settings. The biplot analysis revealed that the B20 environment exhibited the highest proximity to the AEC across all the attributes examined. The findings yielded comparable outcomes of^43^.

#### Genotype ranking: best genotype assessment

**Figure 4:**
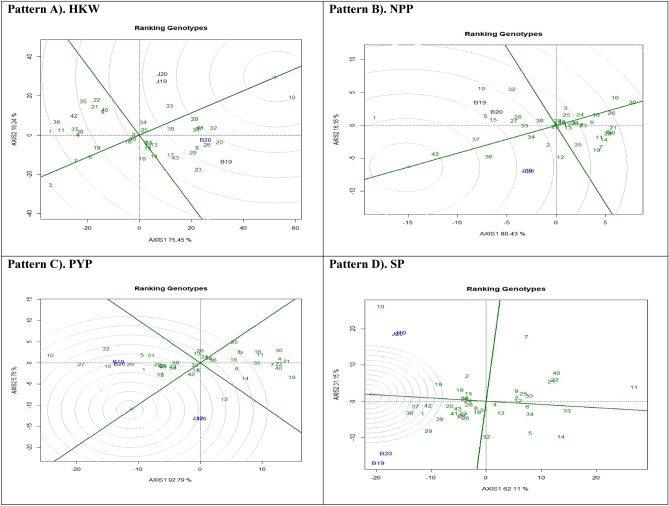
Patterns (A–D). Ranking genotypes based on PC1 and PC2 showing G × E interactions of the 43 groundnut accessions under two locations and two seasons: (**A**) Hundred kernel weight (HKW), (**B**) No. of pods per plant (NPP), (**C**) Pod yield per plant (PYP), (**D**) Shelling percent (SP).

The application of a biplot enabled the determination of optimal and most desirable genotype from a set of 43 genotypes that were assessed. The ideal genotype is consistently located within the middle region and in close proximity to the peak of the arrow within the circular band, as illustrated in Fig. 4. In the instance of HKW (Pattern A), it was noted that genotype G10 (PBS 29079B) was located within the inner circle and considered to be optimal. The genotypes that exhibited close spatial closeness to the inner circle were G32 (PBS 29218) and G28 (PBS 29210). In contrast, it was seen that G3 (PBS 19013) exhibited the greatest distance from the arrowhead in the plot, whereas G1 (Girnar 2) and G7 (PBS 29069) followed suit in terms of their proximity to the arrowhead. In the specific instance of NPP (as illustrated in Fig. 4: Pattern B), it was observed that the genotype G42 (PBS 29230) demonstrated the closest proximity to the ideal genotype, followed by G37 (PBS 29233), G1 (Girnar 2), G36 (PBS 29232), and G30 (PBS 29212). Conversely, genotypes G16 (PBS 29165) and G26 (PBS 29207) were situated further away from the innermost circle. In the context of Pattern C for the pod yield per plant (PYP), it is observed that the inner circle did not exhibit any genotypes. The genotypes in close proximity to the inner circle were G37 (PBS 29233), G2 (Mallika (C2)), G1 (Girnar 2 (C1)), G29 (PBS 29211), and G15 (PBS 29160). In contrast, G30 (PBS 29212), G21 (PBS 29193), and G4 (PBS 19015) were positioned at a substantial distance from the circle representing the best genotypes. Regarding the phenomenon of SP, as illustrated in Fig. 4, Pattern D, it was noted that there were no genotypes located within the inner circle. The genotypes G36 (PBS 29232) and G37 (PBS 29233) were observed in close proximity to the inner circle, but G11 (PBS 29082) and G33 (PBS 29219) were located at a significant distance from it.

For an effective selection, an ideal and best genotype is required to have both high mean and stability properties. A ring at the head of the arrow on the horizontal AEC abscissa axis generally represents an ideal genotype, and additionally, the best genotype should be positioned in the small circle on the AEC abscissa line^20^. Plant breeders used data from agronomic performance during evaluations on the basis of mean performance and stability to choose genotypes best suited to a specific environment within a multi-environment, while genotypes close to the ideal genotype were also more promising or appropriate.

#### Mean vs. stability

**Figure 5:**
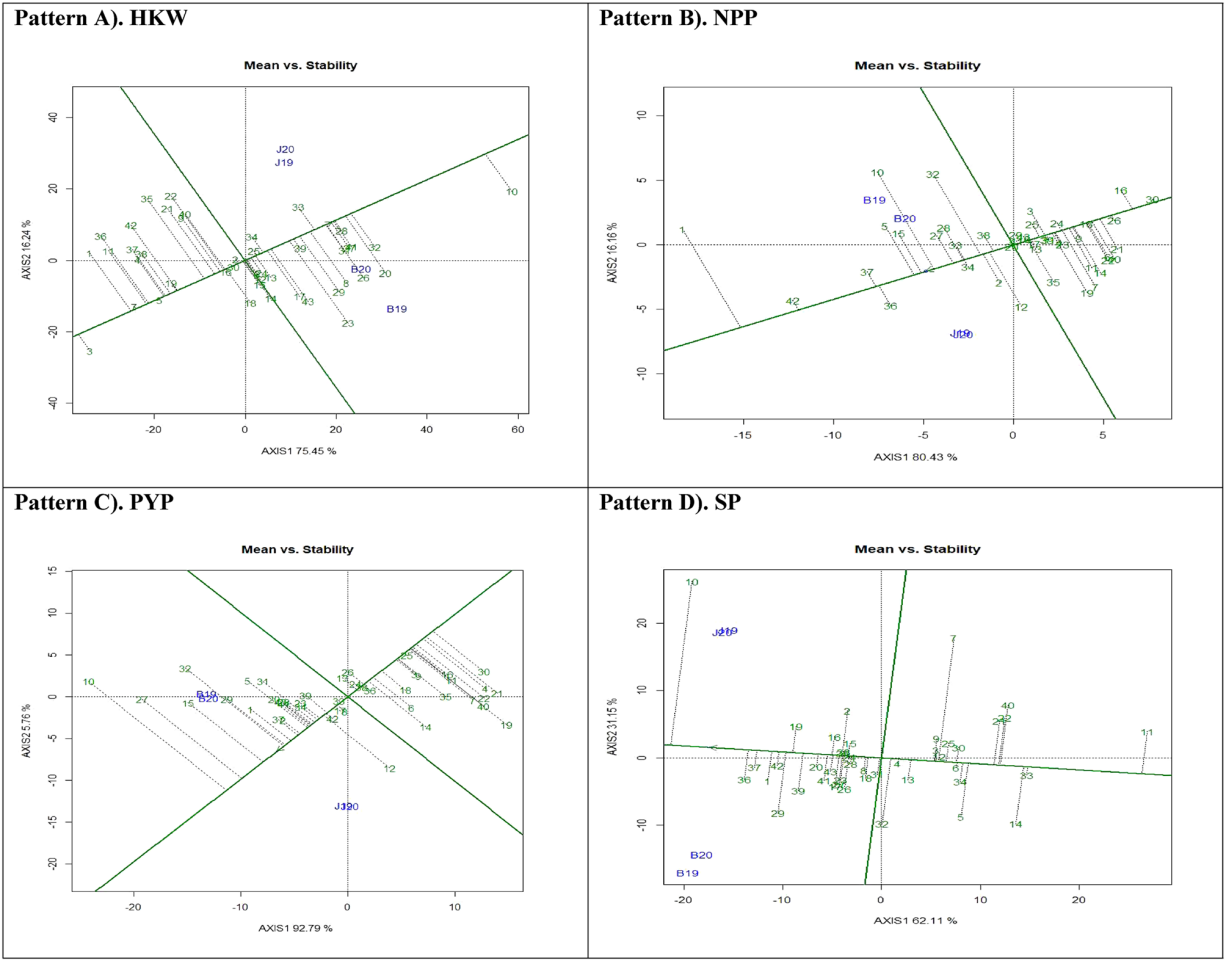
Patterns (A–D). Mean vs. stability based on PC1 and PC2 showing G × E interactions of the 43 groundnut accessions under two locations and two seasons: (**A**) Hundred kernel weight (HKW), (**B**) No. of pods per plant (NPP), (**C**) Pod yield per plant (PYP), (**D**) Shelling percent (SP).

The graphical representation depicted in Fig. 5 displays the mean and stability perspectives of assessed genotypes. The abscissa of the axis of average environment coordination (AEC) is a unidirectional line that traverses the biplot origin and is demarcated by an arrow pointing towards the direction of the most proficient genotypes (i.e., those with the highest level of dormancy). The AEC ordinate axis, which is horizontal in nature, can be defined as the line that traverses through the biplot origin and is perpendicular to the AEC abscissa. The AEC ordinate serves as an approximation of the genotypic contribution to the G × E interaction. Closer the genotype to AEC abscissa, the more consistent or stable under various test environments^44,45^. For HKW (Pattern A) genotypes were partitioned into two groups based on their ordinate values, namely those that produced yields above and below the average. Genotypes, G10 (PBS 29079B), G32 (PBS 29218), G20 (PBS 29191), G41 (PBS 29225), G27 (PBS 29208) and G34 (PBS 29223) exhibited means that were significantly higher than the average means. In contrast, G3 (PBS 19013) succeeded by G1 (Girnar 2), G7 (PBS 29069), and G11 (PBS 29082) exhibit values that are comparatively lower than the mean. Genotypes G25 (PBS 29204), G2 (Mallika), G30 (PBS 29212), G5 (PBS 19018), and G7 (PBS 29069) exhibit the highest degree of stability, while G23 (PBS 29197), G35 (PBS 29228), and G22 (PBS 29194) are deemed to be unstable based on their projection on the abscissa towards the ordinate.

Genotypes G1 (Girnar 2), G42 (PBS 29230), G37 (PBS 29233), G36 (PBS 29232), G5 (PBS 19018), and G15 (PBS 29160) exhibit the highest above-average means for NPP (Pattern B). Conversely, genotypes G30 (PBS 29212), G16 (PBS 29165), G26 (PBS 29207), and G21 (PBS 29193) demonstrate the below-average means. Genotypes, G1 (Girnar 2), G10 (PBS 29079B), and G32 (PBS 29218), were unstable, while G30 (PBS 29212), G18 (PBS 29187), G43 (Raj Mungpali 3), and G20 (PBS 29191) were identified as stable.

In the context of PYP (Pattern C), it was observed that the genotypes G10 (PBS 29079B), G27 (PBS 29208), and G15 (PBS 29160) exhibited the highest mean values. Conversely, genotypes G30 (PBS 29212), G21 (PBS 29193), G4 (PBS 19015), and G22 (PBS 29194) displayed mean values that were below average. The genotypes G10 (PBS 29079B), G32 (PBS 29218), and G19 (PBS 29189) exhibit a high degree of instability based on the projection of AEC ordinate. Conversely, genotypes G42 (PBS 29230), G17 (PBS 29167), G8 (PBS 29078), G33 (PBS 29219), G38 (PBS 29243), and G25 (PBS 29204) demonstrate a high level of stability.

In Pattern D of the SP dataset, PBS 29079B (G10) followed by PBS 29232 (G36) and PBS 29233 (G37) exhibit highest values above the mean, while PBS 29082 (G11) followed by PBS 29219 (G33) and PBS 29143 (G14) exhibit values below the mean. The stability of AEC ordinates G4 (PBS 19015), G24 (PBS 29199), G6 (PBS 19029), and G12 (PBS 29137) has been determined through projections, revealing that they are stable. Conversely, G10 (PBS 29079B), G7 (PBS 29069), G40 (PBS 29195), and G32 (PBS 29218) have been found to be unstable.

The study determined that genotypes G37 (PBS 29233) and G42 (PBS 29230) were desirable as they displayed high pod yield and stability with consistent performance under different environmental conditions, which was in agreement with reports of^28^. The genotypes PBS 29079B (HKW, PWP, SP) and Girnar 2 (NPP) exhibited superior performance, however, their lack of stability suggests that their performance was inconsistent and unpredictable. PBS 19013, Girnar 2 (HKW), PBS 29189 (NPP), PBS 29212 (PWP), and PBS 29082 (SP) were found to exhibit instability and suboptimal performance. Comparable findings were reported by^7,46,47^.

#### BLUP estimated values of 43 genotypes

**Figure 6:**
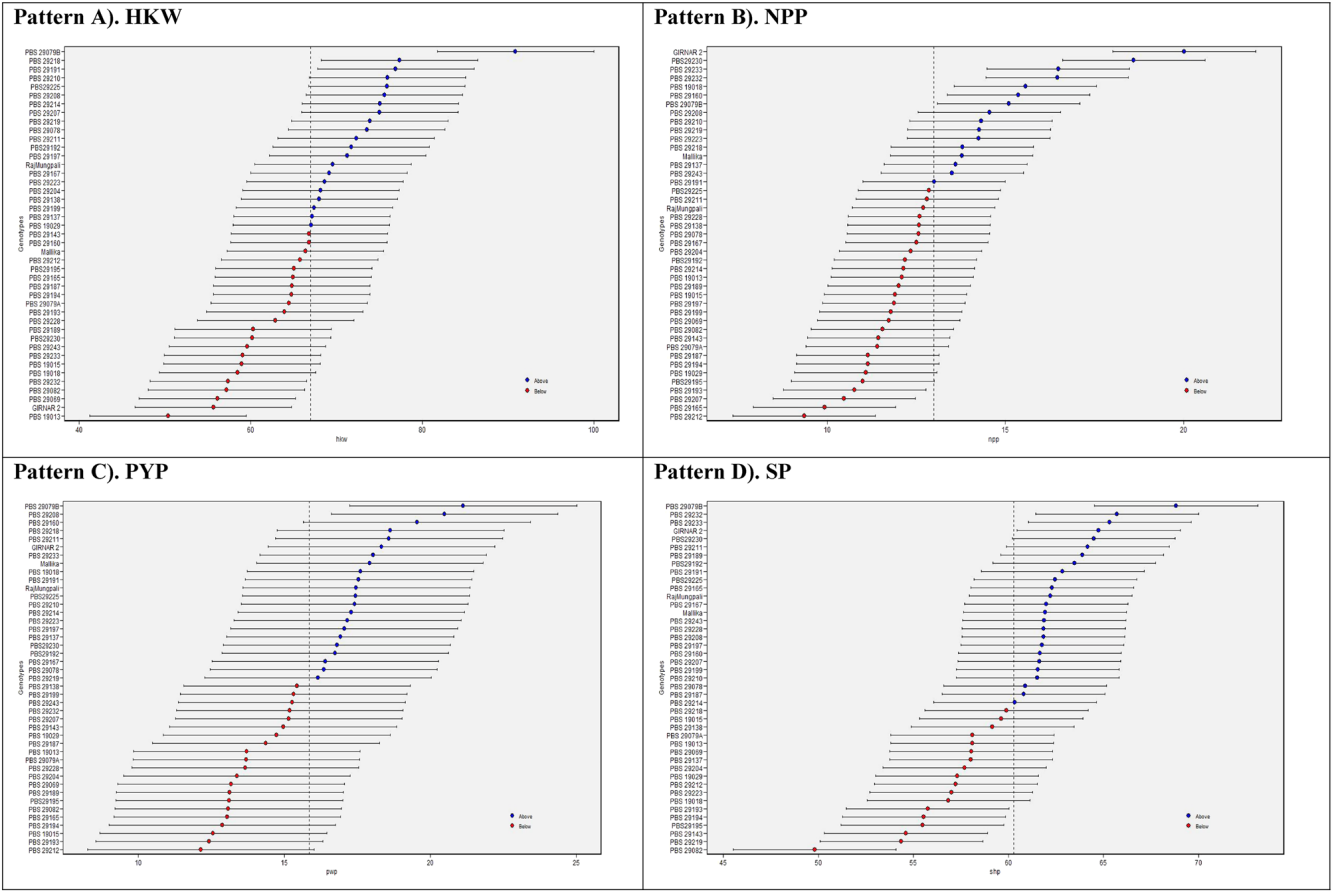
Patterns (A–D). BLUP estimated values for 43 groundnut genotypes: (**A**) Hundred kernel weight (HKW), (**B**) No. of pods per plant (NPP), (**C**) Pod yield per plant (PYP), (**D**) Shelling percent (SP).

Figure 6 presents the Best Linear Unbiased Prediction (BLUP) predicted values for 43 groundnut genotypes, arranged in a descending order. The genotypes with above-average mean performances are represented by blue circles, while genotypes with below-average performances are denoted by red circles. The genotypes positioned at the lowermost section of the graph had the lowest level of proficiency. Figure 6 illustrates the 95% confidence interval for the expected values of HKW, NPP, PYP, and SP, for each genotype. These estimated values are shown by the horizontal error bars. In the context of HKW, PBS 29079B demonstrated the highest mean performance (> 85gm in both locations over seasons), followed by PBS 29218, PBS 29191, and PBS 29210, all of which exhibited above-average mean performance conversely, the mean performances of PBS 19013, Girnar 2, and PBS 29069 were found to be the lowest. The NPP was highest in Giranr 2 followed by PBS 29230 and PBS 29233. Conversely, the lowest average performance was found in PBS 29212, followed by PBS 29165. In the PYP, G10 (PBS 29079B) had highest mean performance closely followed by PBS 29208, PBS 29160, and PBS 29218. On the other hand, PBS 29212 exhibited the lowest performance. In terms of SP, PBS 29079B had the highest average performance, whilst PBS 29082 presented the lowest average performance.

#### Which-won-where

**Figure 7:**
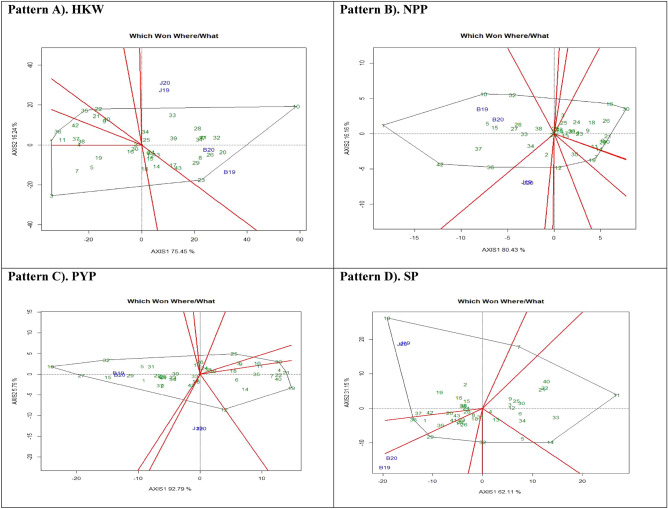
Patterns (**A–D**). Polygon views of GGE biplot for which-won-where analysis of 43 accessions under the effects of genotypes-by environment interactions under two locations and two seasons: (**A**) Hundred kernel weight (HKW), (**B**) No. of pods per plant (NPP), (**C**) Pod yield per plant (PYP), (**D**) Shelling percent (SP).

Use of polygon view of “which-won-where” biplot is a key component of the GGE, which helps to visualize the interaction patterns between genotypes and environments, to show the presence of crossover GEI, mega-environment differentiation, and specific adaptation^23,24,45^. Figure 7 illustrates the polygon view of GGE biplot pattern of Hundred kernel weight (HKW, Pattern A), Number of pods per plant (NPP, Pattern B), Pod yield per plant (PYP, Pattern C) and Shelling percent (SP, Pattern D). First and second interaction components (IPCA1 and PCA2) of G + GE biplot accounted for 91.69%, 96.59%, 98.55%, and 93.26% of the total variation for HKW, NPP, PYP, and SP, respectively. The test environments fell into one of the seven sectors, two of the ten sectors, two of the eight sectors, and two of the seven sectors outlined on the polygon view for HKW, NPP, PYP, and SP, respectively. HKW has one mega environment including both Junagadh and Bikaner, two mega-environments for NPP, PYP, and SP with J19, J20 grouped together in a mega-environment, while B19 and B20 is in the second mega-environment. G10 (PBS 29079B) and G23 (PBS 29197) are the vertex genotype identified for HKW and they are the most responsive to environmental interaction; The most responsive genotypes forming the vertices of NPP were G10 (PBS 29079B), G32 (PBS 29218), G1 (Girnar 2), G42 (PBS 29230) and G36 (PBS 29232) for the first mega environment (B19&B20); G10 (PBS 29079B), G32 (PBS 29218) and G27 (PBS 29208) with one mega environment (B19&B20) and G12 (PBS 29137) with second mega-environment (J19&J20) were the most responsive for PYP; whereas genotypes G29 (PBS 29211) and G10 (PBS 29079B) were more responsive with Bikaner and Junagadh environments for SP. Some vertex genotypes fell into sectors having no test environment, for instance, G3 (PBS 19013), G36 (PBS 29232), G35 (PBS 29228) and G32 (PBS 29218) (Pattern A); G12 (PBS 29137), G19 (PBS 29189), G7 (PBS 29069), G30 (PBS 29212), G20 (PBS 29191) and G16 (PBS 29165) (Pattern B); G19 (PBS 29189), G21 (PBS 29193), G30 (PBS 29212) and G25 (PBS 29204) (Pattern C); G36 (PBS 29232), G32 (PBS 29218), G14 (PBS 29143), G11 (PBS 29082) and G7 (PBS 29069) (Pattern D).

The vertex genotypes identified have superior performance and high adaptability to particular mega environment and are most favoured in those environments^43^. However, vertex genotypes with no environment in the sector are not desirable because of their poor performance across the environments and the genotypes placed within the polygon are less responsive to environment than the corner genotypes^5,19^ The positioning of all environmental indicators into one section of biplot directed that a unique genotype performs best under all tested environments. Oppositely, different genotypes gained in different environments if the environmental indicators were positioned into a different segment of biplot.

## Conclusion

The 43 groundnut accessions used in the current investigation showed different variations in their responses to locations and seasons due to GEI effect and different expressions of genes that regulate the traits. Bikaner location was discriminating and representative and is classified as the superior environment. PBS 29079B was the top most-yielding genotype with highest hundred kernel weight in both locations and in both seasons (85.46gm) and less stable but specifically adapted to Bikaner location. Genotypes, PBS 29079B (HKW, PYP, SP), Girnar 2 (NPP) all performed above average but were not stable, and PBS 19013, Girnar 2 (HKW), PBS 29189 (NPP), PBS 29212 (PWP), and PBS 29082 (SP) were unstable and had below-average yields. The genotypes PBS 29233 and PBS 29230 exhibited beneficial characteristics, including above-average pod yield, hundred kernel weight, number of pods per plant, and shelling percent. Additionally, these genotypes shown great stability across several locations. These genotypes have been identified for cultivation within specific regions or for use as parental lines in confectionary groundnut breeding initiatives.

## Data Availability

All data generated or analysed during this study are included in this published article.

## Abbreviations

AEC: Average environment coordination
AMMI: Additive main effects and multiplicative interaction
GEI: Genotype environment interactions
HKW: 100-Kernel weight
HPS: Hand picked selection
IPCA: Interaction principal component
K: Potassium
N: Total nitrogen
NPP: Number of pods per plant
OC: Organic carbon
P: Accessible phosphorus
PCA: Principal component analysis
PYP: Pod yield per plant
SMK: Sound mature kernels
SP: Shelling percent
SVD: Singular value decomposition

## References

[1] Suchoszek-Łukaniuk, K., Jaromin, A., Korycińska, M. and Kozubek, A. Chapter 103—Health Benefits of Peanut (*Arachis hypogaea* L.) Seeds and Peanut Oil Consumption, Editor(s): Preedy, V.R., Watson, R.R., Patel, V.B., *Nuts and Seeds in Health and Disease Prevention*, Academic Press, Pages 873–880, ISBN 9780123756886 (2011).

[2] Mondal S, Badigannavar AM (2018). Mapping of a dominant rust resistance gene revealed two R genes around the major Rust_QTL in cultivated peanut (Arachis hypogaea L.). Theor. Appl. Genet..

[3] Francisco MLDL, Resurreccion AVA (2008). Functional Components in Peanuts. Crit. Rev. Food Sci. Nutr..

[4] Bishi SK, Lokesh K, Mahatma MK, Khatediya N, Chauhan SM, Misra JB (2015). Quality traits of Indian peanut cultivars and their utility as nutritional and functional food. Food Chem..

[5] Ajay BC, Gowda MVC, Rathnakumar AL, Kusuma VP, Abdul Fiyaz R, Holajjer P (2012). Improving genetic attributes of confectionary traits in peanut (Arachis hypogaea L.) using multivariate analytical tools. J. Agric. Sci..

[6] Kona P, Mahatma MK, Gangadhara K, Ajay BC, Kumar N, Reddy KK (2021). Evaluation and identification of promising advanced breeding lines for quality and yield traits in groundnut (Arachis hypogaea L.). J. Agric. Sci..

[7] Lal C, Ajay BC, Chikani BM, Gor HK (2019). AMMI and GGE biplot analysis to evaluate the phenotypic stability of recombinant inbred lines (RILs) of peanut under mid-season water stress conditions. Indian J. Genet..

[8] Ajay BC, Aravind J, Abdul Fiyaz R, Bera SK, Kumar N, Gangadhar K (2018). Modified AMMI Stability Index (MASI) for stability analysis. ICAR-DGR Newsletter.

[9] Nigam, S.N., Dwivedi, S.L., Reddy, L.J. and Vasudeva Rao, M.J. An update on groundnut breeding activities at ICRISAT centre with particular reference to breeding and selection for improved quality. Proceedings of the Third Regional Groundnut Workshop, held during 13–18 March 1988, Lilongwe, Malwi, 115–25 (1989).

[10] Oladosu Y, Rafii MY, Abdullah N, Magaji U, Miah G, Hussin G (2017). Genotype × Environment interaction and stability analyses of yield and yield components of established and mutant rice genotypes tested in multiple locations in Malaysia. Acta Agric Scand. Sect. B Soil Plant Sci..

[11] Frutos E, Galindo MP, Leiva V (2014). An interactive biplot implementation in R for modeling genotype-by-environment interaction. Stoch. Environ. Res. Risk Assess..

[12] Alizadeh K, Mohammadi R, Shariati A, Eskandari M (2017). Comparative analysis of statistical models for evaluating genotype × environment interaction in rainfed Safflower. Agric. Res..

[13] Amira JO, Ojo DK, Ariyo OJ, Oduwaye OA, Ayo-Vaughan MA (2013). Relative discriminating powers of GGE and AMMI models in the selection of tropical soybean genotypes. Afr. Crop Sci. J..

[14] Fayeun LS, Alake GC, Akinlolu AO (2018). GGE biplot analysis of fluted Pumpkin (*Telfairia occidentalis*) landraces evaluated for marketable leaf yield in Southwest Nigeria. J. Saudi Soc. Agric. Sci..

[15] Alake CO, Ayo-Vaughan MA, Ariyo JO (2015). Selection criteria for grain yield and stability in bambara groundnut (*Vigna subterranean* (L) Verdc) landraces. *Acta Agric*. Scand. Sect. B Soil Plant Sci..

[16] Mndolwa E, Msolla S, Porch T, Miklas P (2019). GGE biplot analysis of yield stability for Andean dry bean accessions grown under different abiotic stress regimes in Tanzania. Afr. Crop Sci. J..

[17] R Development Core Team R: A language and environment for statistical computing. R Foundation for Statistical Computing, Vienna (2022).

[18] De Mendiburu, F. Agricolae: Statistical Procedures for Agricultural Research. Available from https://cran.r-project.org/web/packages/agricolae/index.html (2017).

[19] Bernal, E.F. and Villardon, P.G. GGE Biplot GUI: Interactive GGE Biplots in R. https://cran.r-project.org/web/packages/GGEBiplotGUI/index.html (2016).

[20] Yan W, Kang MS (2003). GGE biplot analysis: A graphical tool for breeders, geneticists, and agronomist (Boca Raton.

[21] Andrade MI, Naico A, Ricardo J, Eyzaguirre R, Makunde GS (2016). Ortiz, R et al Genotype × environment interaction and selection for drought adaptation in sweetpotato (Ipomoea batatas [L.] lam.) in Mozambique. Euphytica.

[22] Chibarabada TP, Modi AT, Mabhaudhi T (2018). Adaptation and productivity of selected grain legumes in contrasting environments of Kwazulu-Natal. South Africa. Int. J. Plant Prod..

[23] Olanrewaju OS, Oyatomi O, Babalola OO, Abberton M (2021). GGE biplot analysis of genotype × environment interaction and yield stability in bambara groundnut. Agronomy.

[24] Khan MMH, Rafii MY, Ramlee SI, Jusoh M, Mamun MA, Halidu J (2021). DNA Fingerprinting, fixation-index (Fst), and admixture mapping of selected bambara groundnut (Vigna subterranea [L.] verdc) accessions using ISSR markers system. Sci. Rep..

[25] Ajay BC, Bera SK, Singh AL, Kumar N, Gangadhar K, Kona P (2020). Evaluation of genotype × environment interaction and yield stability analysis in peanut under phosphorus stress condition using stability parameters of AMMI model. Agric. Res..

[26] Ajay BC, Ramya KT, Abdul Fiyaz R, Govindaraj G, Bera SK, Kumar N (2020). R-AMMI-LM: Linear-fit robust-AMMI model to analyze genotype-by environment interactions. Indian J. Genet..

[27] Ajay BC, Bera SK, Singh AL, Kumar N, Dagla MC, Gangadhar K (2021). Identification of stable sources for low phosphorus conditions from groundnut (Arachis hypogaea L.) germplasm accessions using GGE biplot analysis. Indian J. Genet..

[28] Ajay BC, Abdul Fiyaz R, Bera SK, Kumar N, Gangadhar K, Kona P (2022). Higher Order AMMI (HO-AMMI) analysis: A novel stability model to study genotype-location interactions. Indian J. Genet..

[29] Ajay BC, Kumar N, Kona P, Gangadhar K, Rani K, Rajanna GA (2023). Integrating data from asymmetric multi-models can identify drought-resistant groundnut genotypes for drought hot-spot locations. Sci. Rep..

[30] Narasimhulu R, Veeraraghavaiah R, Sahadeva Reddy B, Tara Satyavathi C, Ajay BC, Sanjana Reddy P (2023). Yield stability analysis of pearl millet genotypes in arid region of India using AMMI and GGE biplot. J. Environ. Biol..

[31] Gangadhara K, Rani K, Ajay BC, Singh S, Kona P, Mori KK (2022). Multi-seasons evaluation of Spanish bunch advanced breeding lines for fresh seed dormancy in groundnut (Arachis hypogaea L.). Ann. Agric. Res. New Series.

[32] Mogale, T.E. Multi-location field evaluation of bambara groundnut (*Vigna subterranean* (L) verdc) for agronomic performance and seed protein, doctoral dissertation (2018).

[33] Esan VI, Oke GO, Ogunbode TO (2023). and Obisesan, IAAMMI and GGE biplot analyses of Bambara groundnut [Vigna subterranea (L.) Verdc.] for agronomic performances under three environmental conditions. Front. Plant Sci..

[34] Gauch, H.G., and Zobel, R.W. AMMI analysis of yield trials *in Genotype-by Environment interaction*, vol. ISBN: 9780849340031. Eds. M. S. Kang and H. G. Jr Gauch (Boca Raton, FL.: Taylor and Francis), 85–122 (1996).

[35] Vargas M, Crossa J (2000). The AMMI analysis and graphing the biplot. biometrics and statistics unit, CIMMYT combining features of AMMI and BLUP techniques. Agron. J..

[36] Yan W, Hunt LA (2001). Interpretation of genotype environment interaction for winter wheat yield in Ontario. Crop Sci..

[37] Kaya Y, Palta C, Taner S (2002). Additive main effects and multiplicative interactions analysis of yield performance in bread wheat genotypes a cross environments. Turk. J. Agric..

[38] Sivapalan S, O'Brien L, Ortiz Ferrara G, Hollamby GJ, Barclay I, Martin PJ (2000). An adaptation analysis of Australian and CIMMYT/ICARDA wheat germplasm in Australian production environments. Crop Pasture Sci..

[39] Voltas, J., Van, E.F., Igartua, E., García del Moral, L.F., Molina-Cano, J.L., Romagosa, I. Genotype by environment interaction and adaptation in barley breeding: basic concepts and methods of analysis. Barley science: Recent advances from molecular biology to agronomy of yield and quality 205 p. (2002).

[40] Purchase, J.L. Parametric stability to describe G x E interactions and yield stability in winter wheat. PhD Thesis, department of agronomy, faculty of Agric.Univ. of Orange Free State, Bloemfontein, South Africa (1997).

[41] Kilic H (2014). Additive main effects and multiplicative interactions (AMMI) analysis of grain yield in barley genotypes across environments. J. Agric. Sci..

[42] Yan W, Kang MS, Ma B (2007). GGE biplot vs AMMI analysis of genotype-by-environment data. Crop Sci..

[43] Hashim N, Rafii MY, Oladosu Y, Ismail MR, Ramli A, Arolu F (2021). Integrating multivariate and univariate statistical models to investigate genotype environment interaction of advanced fragrant rice genotypes under rainfed condition. Sustainability.

[44] Yan W (2001). GGE biplot- a window application for graphical analysis of multi-environmental data and other types of two-way data. Agron. J..

[45] Yan W, Tinker NA (2006). Biplot analysis of multi-environment trial data: Principles and applications. Can. J. Plant Sci..

[46] Shim KB, Shin SH, Shon JY, Kang SG, Yang WH, Sung-GiHeu SG (2015). Interpretation of genotype × environment interaction of sesame yield using GGE Biplot analysis. Korean J. Crop Sci..

[47] Kumar N, Ajay BC, Dagla MC, Rathanakumar AL, Radhakrishnan T, Lal C (2019). Multi-environment evaluation of Spanish bunch groundnut genotypes for fresh seed dormancy. Indian J. Genet..

